# A New Player in the “Synaptopathy” of Alzheimer’s Disease – Arc/Arg 3.1

**DOI:** 10.3389/fneur.2013.00009

**Published:** 2013-02-13

**Authors:** Talitha L. Kerrigan, Andrew D. Randall

**Affiliations:** ^1^School of Physiology and Pharmacology, Faculty of Medical and Veterinary Sciences, University of BristolBristol, UK; ^2^Institute of Biomedical and Clinical Sciences, University of Exeter Medical SchoolExeter, UK

**Keywords:** Arc, amyloid beta protein, Alzheimer’s disease, neurophysiology, AMPA receptor trafficking, intrinsic plasticity

## Abstract

Alzheimer’s disease (AD) is increasingly referred to as a “synaptopathy.” This moniker reflects the loss or damage of synapses that occurs as the disease progresses, which in turn produces functional degeneration of specific neuronal circuits and consequent aberrant activity in neural networks. Accumulating evidence supports the functional importance of the early-expression activity-regulated cytoskeletal (*Arc*) gene in regulating memory consolidation. Interestingly, AD patients express anomalously high levels of Arc protein. Arc physically associates with presenilin1, a pivotal protease for the generation of Amyloid β (Aβ) peptides. Arc expression itself is disrupted in the vicinity of Aβ oligomers and plaques. Such alterations result in the interruption of neuronal network integration *in vivo*. It is not clear what the impacts of these alterations are on the functional neurophysiology of transgenic mouse models of AD-associated amyloidopathy. Our group and others have described alterations to neuronal excitability and thus intrinsic firing within these transgenic mice models. This brief review will emphasize the rising role of Arc and its involvement in neurophysiological alterations of current AD models.

## Introduction

Alzheimer’s disease (AD) appears to be primarily a disorder of synaptic failure (Selkoe, 2002) which is becoming one of the most predictable features in the pathophysiology of the disease. This “synaptopathy” is associated with disruptions in synaptic structure and function, leading to aberrant neural processing and network disruptions.

Early research on AD pathology focused attention on the involvement of the amyloid precursor protein (APP) pathway and the plaques formed by its proteolytic cleavage product Amyloid β (Aβ; Glenner and Wong, 1984; Hardy and Higgins, 1992; Hardy and Selkoe, 2002). Evidence accumulated in recent years has led to the emergence of the soluble, oligomeric Aβ peptide playing a pivotal role in the disruption of synaptic function and thus neuronal network activity (Walsh and Selkoe, 2004, 2007). Although picomolar concentrations of Aβ may play critical physiological roles in synaptic plasticity (Puzzo et al., 2008) and activity-dependent regulation of synaptic vesicle release (Abramov et al., 2009), abnormal accumulations lead to the self-assembly of neurotoxic Aβ oligomers, which interfere with synaptic function and cause neurodegeneration. These alterations in classic neurophysiological processes are believed to be the main substrates of cognitive decline in AD.

Indeed, levels of soluble Aβ oligomers are highly correlated with synaptic dysfunction in AD. It has been possible to monitor the targeting of Aβ oligomers to synapses (Lacor et al., 2004; Deshpande et al., 2009), and to follow the changes in spine morphology and density (Lacor et al., 2007). These synaptic alterations correspond to the best pathological correlate of memory deficits in AD (DeKosky and Scheff, 1990; Terry et al., 1991; Selkoe, 2002), although the exact mechanisms are still unknown. The intriguing targeting of Aβ oligomers to synapses and their disruption brought our focus to one of the genes shown to be vital for memory consolidation and synaptic plasticity, namely the immediate-early gene Arc/Arg3.1 (early-expression activity-regulated cytoskeletal gene, here on referred to as Arc).

Arc is a neuron-specific, post-synaptic protein that is selectively expressed in Ca^2+^/calmodulin-dependent protein kinases II (CaMKII)-positive neurons (Vazdarjanova et al., 2006). Upon activation, Arc is targeted to the post-synaptic density of synaptically active dendritic spines (Lyford et al., 1995; Steward and Worley, 2001; Moga et al., 2004) where it associates with polysomes (Bagni et al., 2000). Arc interacts with endophilin 2/3 and dynamin, contributing to alpha-amino-3-hydroxyl-5-methyl-4-isoxazole-propionate (AMPA) type glutamate receptor (AMPAR) modulation, by enhanced receptor endocytosis (Chowdhury et al., 2006). The Arc-endosome also traffics APP and physically associates with presenilin (PS1), thereby increasing the amount of activity-dependent Aβ generated (Wu et al., 2011). Interruption of the Arc-PS1 interaction prevents activity-dependent increases of Aβ (Wu et al., 2011). The precise signaling cascades involved in Arc transcription are not well defined. For a more comprehensive review on Arc function and signaling, the reader is referred to: Tzingounis and Nicoll, 2006; Miyashita et al., 2008; Bramham et al., 2010; Shepherd and Bear, 2011.

Arc-mediated endocytosis of AMPARs dampens the activity of neuronal networks, enhancing the activity-dependent generation of Aβ (Wu et al., 2011). If Arc-endosome trafficking and resultant activity-dependent generation of Aβ remained unchecked, it will create a positive feedback mechanism in which the synaptic removal of AMPAR will produce a significant loss of dendritic spines and synaptic activity, resulting in synaptic failure, similar to that observed in AD (Hsieh et al., 2006; Shankar et al., 2007; Li et al., 2010).

This brief review will provide an overview of the importance of “synaptopathy” in the pathogenesis of AD, with particular emphasis being placed on the rising role of Arc, in neurophysiology. We aim to provide a critical assessment of the current literature, to address the impact of altered Arc expression on the molecular and cellular mechanisms underlying the functional neurophysiology in transgenic mouse models of AD-associated amyloidopathy.

## Arc and Synaptic Transmission

Processing of information for memory storage requires specific patterns of activity that lead to the modification of synapse structure and eventually to changes in neural connectivity (Lamprecht and LeDoux, 2004; Marrone, 2007). These modifications can be defined as synaptic plasticity, of which, long-term potentiation (LTP) and long-term depression (LTD) are the two main cellular mechanisms that are associated with learning and memory (Bliss and Collingridge, 1993; Kandel, 2001; Malenka, 2003).

Arc was first identified as a hippocampal transcript strongly induced by epileptic seizures and synaptic plasticity-inducing electrical stimulation in the rat hippocampus (Link et al., 1995; Lyford et al., 1995). Arc is not expressed in presynaptic terminals or axons, but it is notable that its mRNA and protein accumulate in dendrites at sites of recent synaptic activity (Steward et al., 1998). The induction of Arc synthesis upon neuronal activation and its localization to active dendrites, make it a prime candidate for investigating the mechanisms underlying learning and memory. The importance of Arc in learning and memory is corroborated in Arc knock-out (KO) animals where loss of the Arc gene results in unusual phenotypic behavior, wherein the animals are able to retain short term memory formation, however long-term memories cannot be formed (Plath et al., 2006). Reduced Arc expression in the hippocampus by infusion of antisense oligodeoxynucleotides interferes with synaptic plasticity and hippocampus-dependent learning and memory (Guzowski et al., 2000).

It has been demonstrated that Arc transcripts are also induced during certain behaviors. The exploration of a novel environment induces Arc expression in a subset of context-activated pyramidal neurons, and can therefore be associated with experience-dependent forms of plasticity (Guzowski et al., 1999; Vazdarjanova and Guzowski, 2004; Gao et al., 2010; Wibrand et al., 2012). The specificity and characteristic time course of Arc mRNA induction can be used to monitor neural circuit activation following behavior episodes, as initially demonstrated by Guzowski et al. (1999, 2001). They were able to detect from Arc RNA *in situ* hybridization studies that in CA1 neurons, it is possible to distinguish between populations of neurons that responded to two different environments and were able to reveal whether the same neuron was activated twice (Guzowski et al., 1999). This unique correlation between RNA expression and neuronal activity levels, allows for Arc mRNA to be used as a tool for the detection of when and where activity in response to learning is being altered. Arc expression itself differs between brain regions and cell types (e.g., CA1 from CA3), suggesting that it plays an important role in detecting changes in neuronal activity in an experience-dependent manner (Kelly and Deadwyler, 2003; Daberkow et al., 2007; Miyashita et al., 2009). Arc is also heavily involved in different forms of synaptic plasticity, however to cover these is beyond the context of this article. We therefore suggest the following reviews (Bramham et al., 2010; Korb and Finkbeiner, 2011; Shepherd and Bear, 2011).

Episodic hippocampal-dependent memory loss, is the earliest clinical sign of AD, and is thought to be a result of changes in synaptic function rather than neuronal loss (Morrison and Hof, 1997; Arendt, 2009). *In vivo* brain imaging studies, using functional magnetic resonance imaging (fMRI) have revealed aberrant networking in brain regions linked to memory function (Sperling et al., 2009). High levels of amyloid deposition are associated with this aberrant default network, suggesting that amyloid pathology in early stages of AD is linked to neural dysfunction, memory loss, and aberrant synaptic plasticity (Sperling et al., 2009). Although still unclear, a general picture is emerging in which Aβ oligomers seem to highjack the molecular machinery necessary to induce synaptic plasticity. It appears that Aβ induces aberrant synaptic plasticity by inhibiting LTP, and more interestingly, by facilitating LTD, causing AMPAR endocytosis (Hsieh et al., 2006; Shankar et al., 2007; Li et al., 2010).

To prevent these imbalances in synaptic plasticity from developing in normal physiology, neurons have developed a unique mechanism which modulates their global levels of post-synaptic AMPAR in response to the level of activity seen in the cells, as expressed by the rate of action potential firing. This process is known as synaptic scaling and is thought to maintain post-synaptic action potential firing rates within certain bounds (average firing rate; Turrigiano et al., 1998). Synaptic scaling is a cell-wide mechanism of plasticity, and is thus referred to as a form of homeostatic plasticity (Fregnac, 1998; Galante et al., 2001). This form of plasticity is particularly sensitive to the levels of Arc (Shepherd et al., 2006; Turrigiano, 2007). Indeed, Arc KO animals as well as neurons overexpressing Arc are not capable of maintaining this negative feedback mechanism (Shepherd et al., 2006).

**Figure 1:**
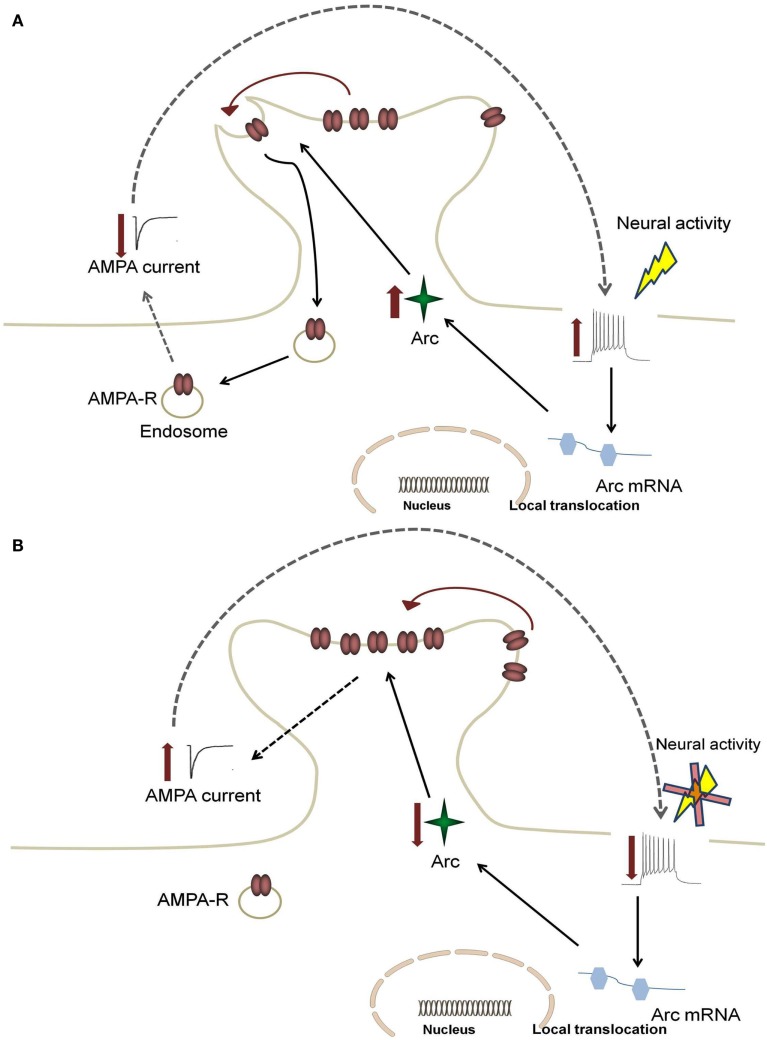
**Feedback mechanism for Arc-mediated AMPA receptor endocytosis**. An increase in neural firing leads to somatic alterations in calcium levels, which causes increased activation in the nucleus to enhance local translocation of Arc mRNA. This increase results in enhanced Arc protein levels and increased AMPA receptor (AMPA-R) endocytosis **(A)**. This leads to a decrease in synaptic strength, which subsequently returns firing rates back to target levels **(B)**. In AD, enhanced Arc expression due to hyperexcitability leads to a positive feedback mechanism in which the dampening response of Arc is limited.

Although the detailed mechanisms through which Arc affects hippocampal functions are still under investigation, recent evidence has demonstrated a role of Arc in AMPAR trafficking, with evidence pointing to regulation of AMPAR endocytosis (Chowdhury et al., 2006; Rial Verde et al., 2006; Shepherd et al., 2006). Arc directly interacts with the endocytic machinery by binding to endophilin 1 and dynamin 2, and selectively increasing the rate of AMPAR recycling (Chowdhury et al., 2006; Shepherd et al., 2006). These studies suggest that when Arc expression is low the steady state of AMPAR trafficking will shift to increase the distribution of AMPAR to the membrane (Shepherd et al., 2006). The inverse is true under conditions of high Arc expression (see Figure 1). Arc is therefore likely important for limiting the level of neuronal excitation since Arc-mediated endocytosis of AMPARs will dampen activity of neuronal networks.

Arc activity is capable of regulating AMPAR endocytosis and both spine size and type. These distinct actions are all means to modulate synaptic strength (Chowdhury et al., 2006; Peebles et al., 2010). As outlined above it appears that Arc controls surface expression of AMPAR in a homeostatic manner and acts to keep surface levels and subunit composition optimal for Hebbian plasticity in normal physiology (Shepherd et al., 2006; Gao et al., 2010).

If Arc-mediated endocytosis remain unchecked, excessive modifications of synaptic strength might generate instability or altered synchrony in neuronal networks, leading in turn to disease states characterized by network imbalances, as observed in AD and epilepsy (Driver et al., 2007; Bragin et al., 2009; Palop and Mucke, 2009). The AD-associated peptide, Aβ, depresses AMPA receptor currents in brain slices and induces AMPAR endocytosis via a mechanism which is similar to Group 1 metabotropic glutamate receptor (mGluR-LTD; Hsieh et al., 2006). There is also further evidence that AMPAR trafficking is reduced in certain transgenic mouse models overexpressing APP (Almeida et al., 2005; Chang et al., 2006). Is it therefore plausible that AD may follow a similar mechanism in which a disruption of the Arc protein and its expression results in aberrant AMPAR trafficking?

## Functional Neurophysiology in AD Models – A Rising Role for Arc?

A number of compelling findings suggest that Arc may contribute to the cognitive deficits and Aβ-dependent alterations in synaptic plasticity experienced in AD. In fact, oligomeric forms of Aβ have been shown to induce Arc expression itself (Lacor et al., 2004). The synaptic binding of Aβ was able to induce a sustained Arc expression within minutes, leading to ectopic protein diffusion throughout the dendrite (Lacor et al., 2004).

Intriguingly, some AD mouse models show a decrease in the number of Arc-expressing cells in the cortex (Wegenast-Braun et al., 2009), a reduced Arc mRNA expression following explorative behavior (Palop et al., 2005; Wegenast-Braun et al., 2009), and lower levels of Arc mRNA in Aβ-containing brain regions (Dickey et al., 2004). Taken together, these data suggest that the widespread Arc expression from acute Aβ exposure might be paralleled by down-regulated Arc signaling in transgenic animals which continuously over-produce Aβ. Early-stage synaptic deterioration may be explained by the age-dependent decreases in Arc mRNA and therefore altered dendritic transport in some transgenic mice (Dickey et al., 2003).

Along with the disruption in AMPAR trafficking, there may be structural modifications in the architecture of the neurons taking place, which could account for some of the neurophysiological alterations experienced in AD (Jacobsen et al., 2006; Middei et al., 2008). Indeed, post-mortem tissues from AD patients reveal reduced spine density (Scheff et al., 1996). Several *in vitro* studies have demonstrated that oligomeric Aβ causes a reduction in the number and/or length of dendritic spines in hippocampal neurons (Calabrese et al., 2007; Lacor et al., 2007; Shankar et al., 2007). Similar to Arc KO mice, mouse AD models display similar decreases in spine density and impairment of long-term memory (Jacobsen et al., 2006; Peebles et al., 2010; Perez-Cruz et al., 2011).

A number of studies in mutant APP-expressing transgenic models have indicated increased Arc activation in response to neuronal activity (Grinevich et al., 2009; Perez-Cruz et al., 2011), as shown in Table 1. In these studies the APP-based transgenic lines were studied prior to plaque deposition, suggesting that soluble oligomeric, rather than deposited fibrillar Aβ is responsible for the enhanced Arc expression (Perez-Cruz et al., 2011). In fact, in the Tg2576 mouse line, the observed loss in dendritic spines were said to be attributed to a loss of inhibitory interneurons, which resulted in hyperexcitability caused by enhanced glutamate and calcium-mediated excitation, subsequently causing the enhanced expression of Arc (Perez-Cruz et al., 2011). We have confirmed the hippocampal network hyperexcitability described in this particular mouse line, along with a double mutant APP_SWE_/PS1_M146L_ (PSAPP) mutation and have found that hippocampal CA1 pyramidal cells have enhanced “burstiness” (Brown et al., 2011). Such alterations in excitability could lead to the early disruption in synchronous network activity (Brown et al., 2005; Driver et al., 2007).

**Table 1:** **Summary of effects of AD-related pathologies on *Arc* expression**.

Species	AD related model	Level of Arc expression	Brain region	Reference
Mouse	APP/PS1, APPDutch, and APP23 (aged and young)	Decreased	Hippocampus and neocortex	Wegenast-Braun et al. (2009)
Mouse	APP/PS1	Decreased	Hippocampus	Dickey et al. (2003, 2004)
Mouse	hAPP (preplaque, FAD)	Decreased	Hippocampus (DG) and cortex	Palop et al. (2005)
Human	Neurons with NFT	Decreased	Hippocampus	Ginsberg et al. (2000)
Mouse	Synthetic Aβ	Decreased	Cortical primary cultures	Echeverria et al. (2007)
Rat	Synthetic Aβ	Decreased	Cortical primary cultures	Wang et al. (2006), Chen et al. (2009)
Mouse	TG2576 and APP/Lo (preplaque)	Increase	Hippocampus	Perez-Cruz et al. (2011)
Mouse	APP/PS1 (preplaque)	Increase	Hippocampus	Grinevich et al. (2009)
Mouse	Synthetic Aβ	Increase	Cortical primary cultures	Wu et al. (2011)
Rat	Synthetic Aβ	Increase	Hippocampal primary neurons	Lacor et al. (2004)
Mouse	4–7 Month old hAPP-J20 (preplaque)	Both increase and decrease	Hippocampus	Palop et al. (2005)
Mouse	TG2576 (aged)	No change	Hippocampus	Cuadrado-Tejedor et al. (2011)
Mouse	CRND8 (aged)	No change	Hippocampus	Herring et al. (2012)

Although the increase in cellular excitability described above occurs in the absence of changes to resting potential, it seems to arise from alterations to voltage-gated Na^+^ channels (Brown et al., 2011; Randall et al., 2012). These alterations in Na^+^ channels are an age-dependent event that was absent from the early preplaque Tg2576 mice (used in Perez-Cruz et al., 2011; Brown et al., 2011), however present in the more aggressive Aβ generating PSAPP mouse (Brown et al., 2011). We cannot rule out the involvement of a possible bidirectional control of intrinsic excitability (Fan et al., 2005; Brager and Johnston, 2007), as a result of enhanced neuronal activity due to increased Aβ burden.

Interestingly, a recent study revealed that Arc increases the association of presenilin/γ-secretase with endosomes that traffic APP (Wu et al., 2011). When binding of Arc to PS1 was interrupted activity-dependent increases in Aβ ceased. These workers also revealed that the level of Arc expression determines the burden of Aβ in the APP-based model used. This was the first study to reveal that Arc protein was capable of increasing the generation of Aβ *in vivo*. Wu et al. (2011) further explored the role of Arc in AD by examining the medial frontal cortex of post-mortem human tissue of patients with AD. The levels of Arc protein were significantly increased in the medial frontal cortex of patients with late stage advanced AD (Braak Stage V and VI) when compared to their non-demented age-matched controls. Based on this the authors concluded that Arc expression could contribute to Aβ generation and pathology in AD (Wu et al., 2011).

Another *in vivo* study revealed that amyloid plaques act locally to aberrantly increase Arc expression in active neurons (Rudinskiy et al., 2012). However, the proportion of neurons that were active in the location of amyloid plaques was significantly decreased. This variation in active neurons near pathological plaques could provide an explanation to the discrepancies that exist in current literature involving Arc expression in AD (Table 1). Moreover this study provides valuable insight into the underlying mechanisms which could contribute to the alterations experienced in the “default mode network, DMN” (Raichle et al., 2001) of patients with AD, which is currently being explored in clinical neurophysiology. fMRI studies and position emission tomography (PET) of AD patients have revealed a distinct correlation between default activity patterns in cortical regions in young adults prior to the development of AD and topography of Aβ deposition in early AD cases (Buckner et al., 2005; Sperling et al., 2009). This correspondence raises the possibility of a relationship between activity patterns in early adulthood and later Aβ deposition, providing valuable information and possible insight into the development of AD. Indeed, Rudinskiy et al. (2012) suggest that the pattern of Arc expression reflects the nervous system responses to, and physiological consolidation of, behavioral experience. They conclude that disruptions in Arc patterns reveal plaque-associated interference with neural network integration, which could ultimately lead to the synaptopathy of AD.

## Concluding Remarks

Despite its well characterized role in synaptic plasticity, Arc’s involvement in disease is less well understood. It appears that this enigmatic protein plays an imperative role in the maintenance of homeostatic neuronal activity, which if disrupted, presents itself in pathology. In this review, we focused on some of the most valuable findings, providing a unique insight into the mechanisms underlying cognitive decline associated with AD.

We have explored the role of Arc in different models of AD-associated pathology, and experienced a range of fluctuations in neurophysiological properties. Differences *in vitro* can be interpreted as the result of a number of variables that differ between each of the reported studies, including differences in behavioral assays performed. Not only do they include different brain regions, they also include different strains and ages of mice studied. Differences in genetic background and gene inclusions can themselves have profound effects on neurophysiology. Although a detailed morphological analysis of changes in synaptic connectivity in the AD brain over time is difficult and limited to the use of post-mortem material, the generation of APP transgenic mice that over-produce Aβ has enabled a better understanding of the functional and morphological consequences of Aβ overproduction. These studies further highlight the need to understand precisely how Arc expression is affected in AD, and its consequent alterations to neurophysiological function. Valuable evidence now emerging from *in vivo* models can directly be correlated to clinical studies. This provides further support for the continuation of exploring the role of Arc in the synaptopathy of AD. Finally, improving our understanding of the molecular mechanisms contributing to maintaining or strengthening synapses may be an interesting entry point for novel therapeutic intervention in many neurodegenerative diseases.

## Conflict of Interest Statement

The authors declare that the research was conducted in the absence of any commercial or financial relationships that could be construed as a potential conflict of interest.
